# From Single-Modal Signal Recognition to Multidimensional Precision Detection: Breakthroughs and Innovations of Multi-Modal Sensors in Tumor Biomarker Detection

**DOI:** 10.3390/bios16100579

**Published:** 2026-10-15

**Authors:** Ruimiao Chang, Jingjing Wang, Yong Li, Shuai Jia, Liping Li, Ruiping Zhang

**Affiliations:** 1 Shanxi Bethune Hospital, Shanxi Academy of Medical Sciences, Third Hospital of Shanxi Medical University, Tongji Shanxi Hospital, Taiyuan 030032, China; changruimiao@sxbqeh.com.cn (R.C.); liyong@sxbqeh.com.cn (Y.L.); 2 School of Pharmacy, Shanxi Medical University, Taiyuan 030001, China; 18739453696@163.com (J.W.); aaronchiac5@163.com (S.J.); 3 Key Laboratory of Cellular Physiology, Shanxi Medical University, Ministry of Education, Taiyuan 030001, China; 4 Medicinal Basic Research Innovation Center of Chronic Kidney Disease, Shanxi Medical University, Ministry of Education, Taiyuan 030001, China; 5 School of Basic Medical Sciences, Shanxi Medical University, Taiyuan 030001, China; 6 The Radiology Department, Shanxi Provincial People’s Hospital Affiliated to Shanxi Medical University, Taiyuan 030001, China

**Keywords:** tumor biomarkers, early diagnosis, single-modal sensors, multi-modal sensors, signal cross-validation, precision diagnostics

## Abstract

The sensitive detection of tumor markers is of great significance for clinical diagnosis. Due to the extremely low abundance of early tumor biomarkers and severe background interference from biological matrices, traditional single-modal atomolar biological testing generally has limitations such as high false positives and narrow linear ranges, making it difficult to meet the clinical needs of precise diagnosis and treatment. This has driven the iterative development of biosensing technology from a single signal readout mode to multi-modal collaborative analysis, significantly improving the sensitivity and accuracy of complex biological sample detection. Dual-modal sensors integrate two complementary sensing mechanisms, whereas multi-modal sensors produce three or more independent signal outputs. By combining such mechanisms, for example electrochemistry and optics, these platforms can establish a signal cross-validation and self-calibration detection system. This multidimensional analysis strategy can mitigate interference caused by environmental factors and human operational errors to a certain extent, lowering detection limits and broadening the linear range, thereby potentially providing higher-reliability data support for early tumor diagnosis. This work systematically reviews the technological evolution and performance differences in tumor biomarker sensors from single-mode to multi-mode in recent years, with a focus on summarizing the cutting-edge research progress in this field. It provides a comprehensive reference for researchers and facilitates further applications of multi-modal sensors in point-of-care detection, and artificial intelligence-assisted analysis.

## 1. Introduction

**Figure 1:**
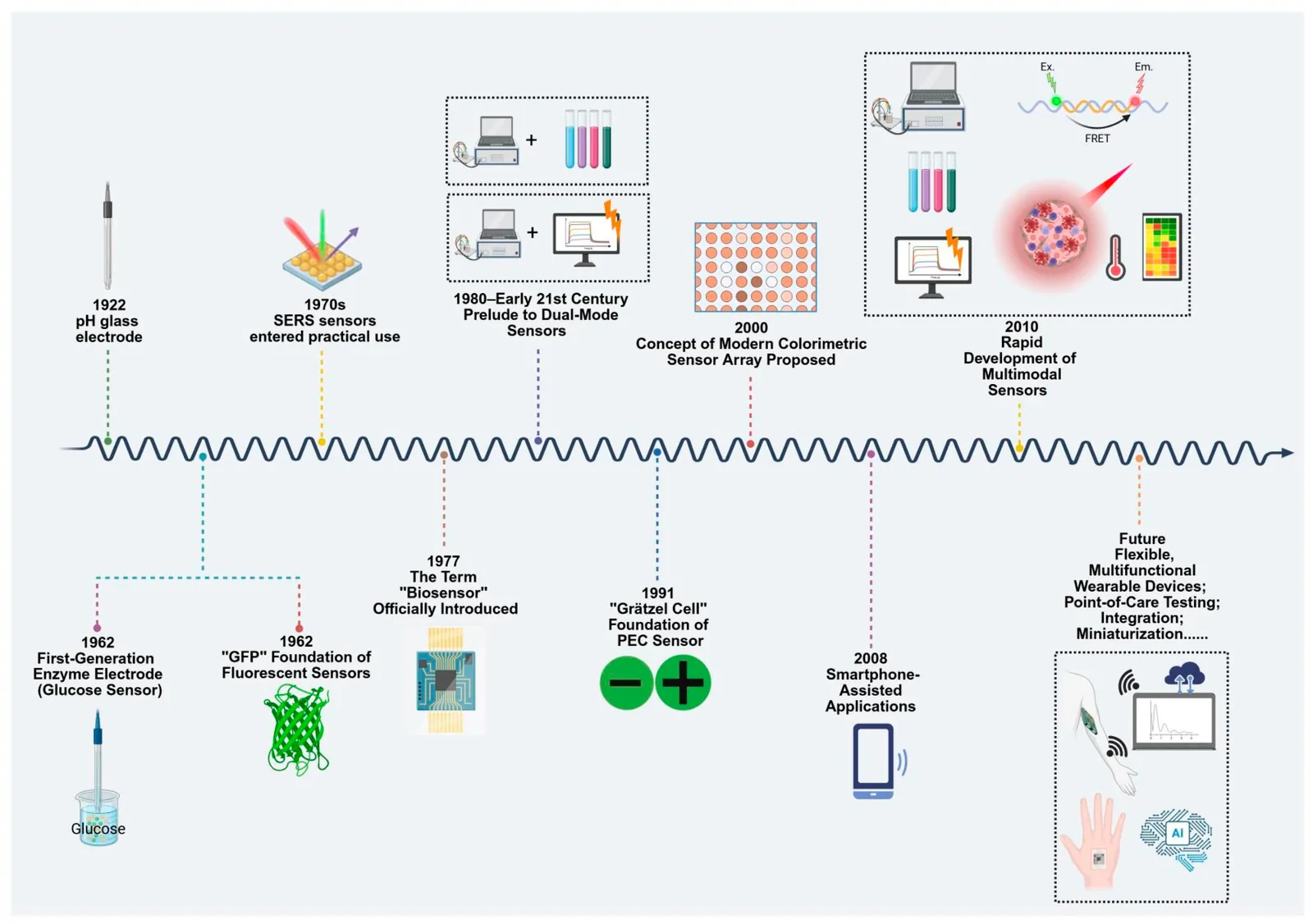
Schematic illustration of the evolutionary process of biosensor development.

In the realm of early cancer diagnostics, biosensors [1,2,3,4,5] represent a highly promising molecular detection strategy, with their developmental trajectory illustrated in Figure 1. Compared to traditional methods reliant on morphological changes, detection systems targeting tumor biomarkers can capture molecular signatures of tumorigenesis at a much earlier stage [6,7,8,9]. Unlike conventional biochemical analyses that require large-scale instrumentation and cumbersome procedures, biosensors integrate biorecognition elements with physicochemical transducers to convert molecular interactions into quantifiable optical or electrical signals. Notably, electrochemical and optical biosensors based on nanomaterials have successfully pushed the limit of detection (LOD) down to the femtomolar (fM) or even attomolar (aM) range, providing powerful tools for capturing low-abundance biomarkers during the incipient stages of cancer [10].

Initially, research efforts were predominantly focused on single-modal sensing, which relies on one signal-transduction mechanism. These platforms have demonstrated unique advantages and achieved significant milestones in specific application scenarios. However, single-modal sensing is inherently susceptible to interference from complex sample matrices, fluctuations in environmental conditions, and operational variations between different personnel, which collectively limit detection accuracy in real biological samples. To address these challenges, researchers have explored the integration of multiple detection modes into a single sensing platform, leading to the development of dual-modal and multi-modal biosensors [11,12,13,14,15,16,17,18,19,20,21,22]. Dual-modal sensing integrates two orthogonal or complementary transduction mechanisms on one platform, and multi-modal sensing produces three or more independent signal outputs. This strategy establishes a unique framework for signal cross-validation and self-calibration. Such synergistic mechanisms not only broaden the linear response range by leveraging the complementary strengths of diverse signals but also partially mitigate background interference through dual-confirmation mechanisms, thereby achieving multidimensional precision in tumor biomarker capture [23,24,25].

This review systematically outlines advances in tumor biomarker detection, which have evolved from single-signal readout strategies to multidimensional synergistic analytical modes. A literature search was performed in Web of Science and Google Scholar, focusing mainly on publications from 2020 to 2026. The search terms included “Tumor biomarkers”, “Early diagnosis”, “Electrochemical sensors”, “Colorimetric sensors”, “Fluorescence sensors”, “SERS sensors”, “PEC sensors”, “Dual-modal sensors”, and “Multi-modal sensors”. The inclusion criteria were original research or review articles published in peer-reviewed journals that described sensing strategies for tumor biomarkers and reported explicit sensing performance parameters, such as the target biomarker, linear range, limit of detection, selectivity, construction time, and sensing modality. We provide a comprehensive summary of the current status of single-modal sensors based on various signal transduction mechanisms, including electrochemistry (EC), colorimetry (CL), fluorescence (FL), surface-enhanced Raman scattering (SERS), and photoelectrochemistry (PEC). Building on this, we focus on the construction principles, signal amplification strategies, and performance advantages of dual-modal sensing platforms, such as EC-CL, EC-FL, EC-SERS, and EC-PEC, as well as multi-modal platforms that produce three or more independent signal outputs. Finally, we discuss the technical challenges associated with miniaturization, multiplexed detection, and intelligent data processing, while offering future perspectives on the application of multi-modal sensors in point-of-care testing (POCT) and precision medicine.

## 2. Tumor Biomarkers

Tumor biomarkers mainly include nucleic acids, proteins, exosomes, and circulating tumor cells (CTCs) [26,27,28]. These biomarkers generally exhibit ultra-low abundance, high heterogeneity, and considerable susceptibility to matrix interference in complex body fluid environments. Among nucleic acid biomarkers, circulating tumor DNA (ctDNA) carries tumor-specific mutations or methylation signatures but suffers from an extremely short half-life and ultralow concentration in blood, where it is frequently masked by abundant normal cellular DNA [29,30,31]; Meanwhile, microRNAs (miRNAs) are highly vulnerable to RNase degradation due to their short sequences and high homology [32]. Accordingly, achieving single-base resolution and robust signal amplification for trace nucleic acid targets remains a core challenge for high-precision nucleic acid sensing.

Protein biomarkers (e.g., carcinoembryonic antigen (CEA), carbohydrate antigen 125 (CA125), alpha-fetoprotein (AFP)) [33,34,35,36,37,38] commonly display aberrant expression during tumorigenesis driven by genetic mutations or post-translational modifications. Although the detection techniques for protein markers are relatively mature, individual biomarkers often lack sufficient diagnostic specificity, and their concentration variations at early tumor stages are exceedingly subtle in peripheral blood [39]. Such limitations have driven sensing strategies toward multiplexed analysis, raising stringent demands for high-throughput, interference-free capture of multiple proteins on a single interface.

Exosomes are 40–150 nm lipid bilayer vesicles that encapsulate abundant tumor-derived molecular information. Their inherent membrane structure protects internal cargoes from enzymatic degradation, endowing exosomes with superior stability in biofluids [40,41,42,43]. However, abundant lipoproteins and protein aggregates in complex matrices render traditional isolation methods time-consuming and of limited purity [44,45], making the development of integrated isolation-and-detection sensing platforms a research focus.

CTCs are shed from solid tumors into body fluids and serve as metastatic “seeds” for tumor progression, providing a non-invasive approach for cancer diagnosis and personalized immunotherapy. However, their extremely low abundance in peripheral blood (approximately 1–10 CTCs per billion blood cells) severely limits capture efficiency [46,47,48,49]. Traditional morphological detection methods are prone to false-negative results, whereas nanostructure-based multi-modal sensing strategies (e.g., magnetic enrichment coupled with SERS fingerprinting) can potentially reduce false-negative rates and enable precise CTC identification [50,51,52].

Collectively, the distinct differences in abundance, inherent stability, and anti-interference capacity among different tumor biomarkers expose the inherent limitations of single-modal sensing in complex clinical samples, strongly motivating the development of dual-modal and multi-modal synergistic sensing strategies.

## 3. Progress in Single-Modal Sensors for Tumor Biomarker Detection

### 3.1. Electrochemical Sensors

Capable of highly sensitive and selective analysis of target analytes [53], electrochemical biosensors are among the most extensively investigated sensing platforms. When bioreceptors bind specifically with target substances on the electrode surface, pronounced changes occur in the interfacial electrochemical signals; these variations are then translated into measurable electrical signals by electrochemical transducers for quantitative detection of target compounds. Various electrochemical techniques are employed for signal acquisition, including cyclic voltammetry (CV) [54], differential pulse voltammetry (DPV) [55], electrochemical impedance spectroscopy (EIS) [56], and square wave voltammetry (SWV) [57].

**Figure 2:**
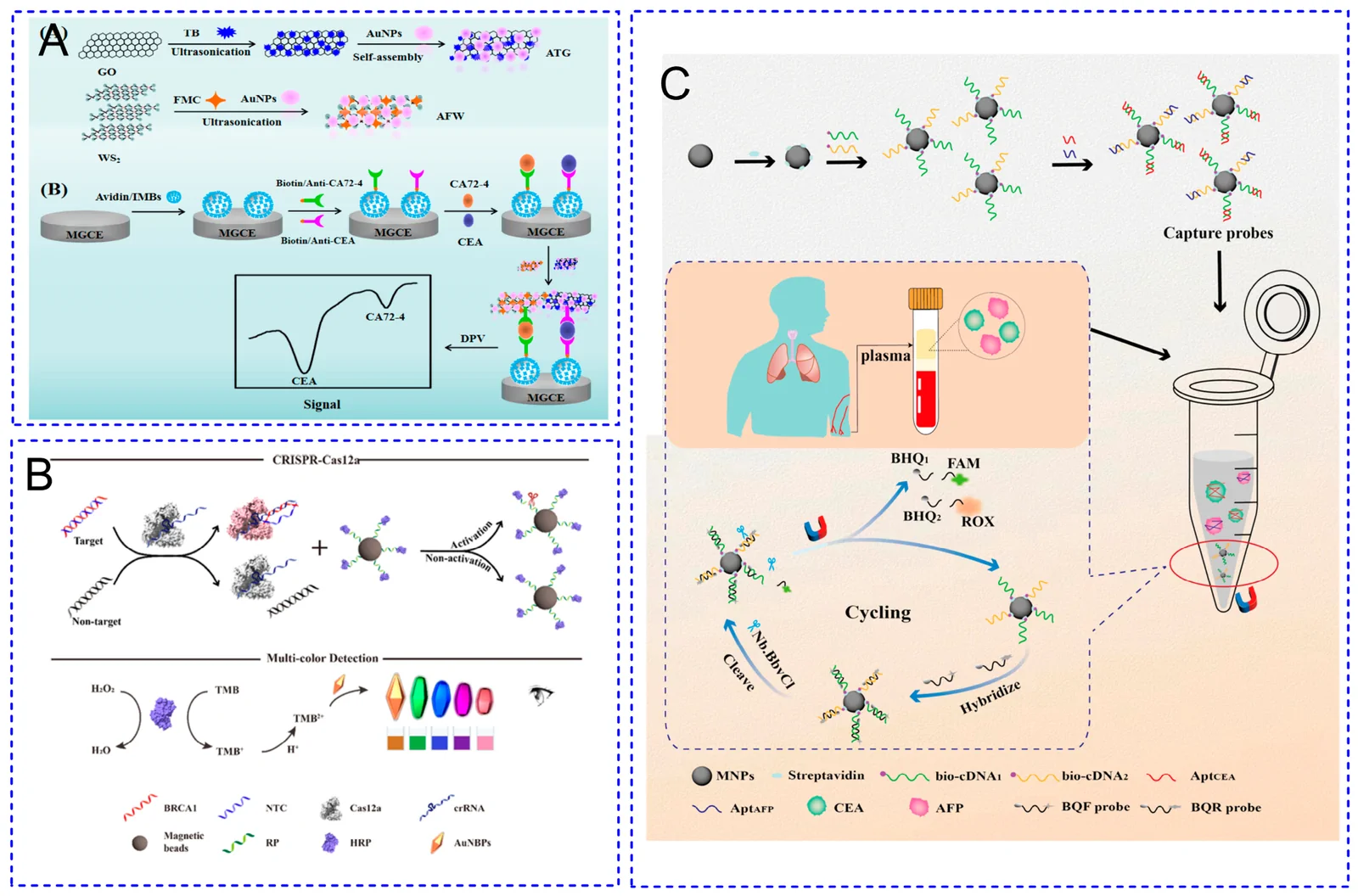
(**A**) Schematic illustration of the synthesis of ATG and AFW nanocomposites and a schematic of the sensor for the simultaneous detection of CEA and CA72-4. Reprinted with permission from ref. [62]. Copyright 2025, Biosensors. (**B**) Schematic illustration of the multicolor visual perception strategy based on CRISPR/Cas12a. Reprinted with permission from ref. [73]. Copyright 2024, Analytical Chemistry. (**C**) Schematic of a “one-pot” fluorescence biosensing platform for the simultaneous detection of CEA and AFP. Reprinted with permission from ref. [87]. Copyright 2025, Talanta.

To overcome the sensitivity bottlenecks associated with traditional electrodes in detecting ultra-trace biomarkers, researchers have extensively utilized highly conductive or catalytically active nanomaterials to engineer the sensing interface. For instance, gold nanoparticles (AuNPs) are frequently employed in sandwich-type configurations due to their exceptional electron transfer rates and biocompatibility [58]. Qin et al. [59] constructed a sandwich structure using AuNPs and amphiphilic polymers to modify the sensing interface, significantly enhancing the capture efficiency of CEA. Wang et al. [60] prepared well-dispersed spherical AuNPs via a green synthesis method using peppermint extract; these nanoparticles were used to modify glassy carbon electrodes (GCE), markedly improving electrochemical performance. Que et al. [61] utilized three-dimensional (3D) cadmium-cobalt oxide nanostructures modified with AuNPs as a nanohybrid platform for the efficient detection of Mucin 1 (MUC1) via DPV. Su et al. [62] developed nanocomposites based on AuNPs-toluidine blue-graphene oxide and AuNPs-ferrocene carboxylic acid-tungsten disulfide to simultaneously label CEA and carbohydrate antigen 72-4 (CA72-4) antibodies. These formed distinct electrochemical signal probes, as illustrated in Figure 2A, and were magnetically immobilized on a magnetic GCE to achieve high-sensitivity, wide linear range dual-target detection. Furthermore, metal–organic frameworks (MOFs) and two-dimensional materials (e.g., MXenes, graphene) have been introduced into electrode modification layers. For example, Li et al. [63] employed ZnS@MSN structures as signal amplification carriers to achieve highly sensitive detection of the pancreatic cancer biomarker carbohydrate antigen 19-9 (CA19-9).

With the burgeoning demand for personalized medicine, electrochemical sensors are evolving toward flexible and wearable architectures [64,65]. Xu et al. [66] developed a wearable microneedle patch based on carbon nanotubes for the real-time monitoring of cytokine levels in interstitial fluid. This innovation integrates sampling, detection, analysis, and communication into a single miniature patch, providing a new pathway for translating laboratory research into clinical applications. Such non-invasive monitoring modes offer new possibilities for post-operative home-based surveillance. Integrated with artificial intelligence, this technology is driving tumor screening toward precision, dynamism, and accessibility at home, offering a revolutionary approach to early cancer diagnosis.

### 3.2. Optical Sensors

Optical sensors utilize light to translate the interactions between bioreceptors and target analytes into visible or measurable optical signals directly related to the analyte concentration. Like their electrochemical counterparts, optical biosensors employ bioreceptors for specific binding, while their transducers utilize techniques such as fluorescence or surface plasmon resonance to detect changes in optical properties [67]. Optical sensors are mainly classified into fluorescent, colorimetric, and SERS sensors. Unlike electrochemical biosensors, they rely on an optical detection principle that monitors variations in optical signals including light absorption, emission and scattering, which enables non-contact measurement with relatively weak background noise.

#### 3.2.1. Colorimetric Sensors

Colorimetric sensors enable the qualitative or quantitative analysis of targets through the intensity of color signals, making them a preferred strategy in the POCT field [68]. These sensors primarily rely on enzymes or nanozymes to catalyze substrate color changes, which can be interpreted directly by the naked eye or via a smartphone. Utilizing multiple chromogenic substrates to detect a single component can compensate for the lack of obvious changes in a single substrate, thereby improving detection sensitivity.

Localized surface plasmon resonance (LSPR) is a core mechanism of colorimetric sensing. The optical properties of AuNPs depend not only on their size but also heavily on their aggregation state. The transition between aggregation (red to purple or blue) and de-aggregation (purple to red) of AuNPs, driven by plasmonic coupling, results in color changes suitable for detecting various analytes. The LSPR characteristics make AuNPs classic chromogenic probes [69]. Harnessing this property, Munyayi et al. [70] achieved label-free detection of the MUC1 protein using aptamer-induced AuNPs aggregation. Bordbar et al. [71] developed a colorimetric paper-based electronic tongue composed of bi-functionalized AuNPs for the simultaneous detection of four tumor biomarkers. Furthermore, the excellent biocompatibility of AuNPs facilitates their conjugation with various biological macromolecules. Tunali et al. [72] developed a novel peptide nucleic acid (PNA)-mediated colorimetric sensing method. Using unmodified AuNPs and specific hybridization between PNA and target DNA, they achieved rapid, label-free detection of the E545K mutation in the PIK3CA gene based on solution color changes.

Moreover, the introduction of the clustered regularly interspaced short palindromic repeats-Cas12a (CRISPR-Cas12a, formerly Cpf1) system has further enhanced the specificity of colorimetric methods. Zhao et al. [73] reported a novel multicolor visual biosensing strategy based on CRISPR/Cas12a, as shown in Figure 2B. By leveraging the trans-cleavage activity of CRISPR/Cas12a to activate magnetic beads and horseradish peroxidase-modified reporter probes, they achieved rapid screening for BRCA1 gene mutations. This method can detect BRCA1 at concentrations as low as 30 pM within 60 min and demonstrated satisfactory performance in human serum samples, showcasing its potential in sensitive biomarker detection and POCT applications. However, it is noteworthy that single-modal colorimetry can be susceptible to interference from the sample’s inherent color (e.g., hemolytic samples), which may reduce sensitivity—a challenge that warrants further attention for future practical applications.

#### 3.2.2. Fluorescence Sensors

Fluorescence sensing represents one of the most promising analytical methodologies currently available [74]. The phenomenon occurs when electrons in a molecular system are excited to higher energy orbits upon irradiation with light of a specific wavelength. Owing to the instability of the excited state, electrons relax back to the ground state and dissipate surplus energy in the form of light, a physical process dominated by the principles of electronic transition [75,76]. Compared to colorimetry, fluorescence sensors offer significantly enhanced sensitivity. Current research focuses on the design of fluorescence probes and the construction of sensing and imaging platforms [77,78], where the versatile functionalization of these probes allows for continuous performance optimization across various biomarker targets.

While traditional fluorophores, such as organic dyes or proteins [79], are prone to photobleaching and potential cytotoxicity, novel probes such as quantum dots (QDs) [80] and upconversion nanoparticles (UCNPs) [81] have become research focal points. Sousa et al. [82] developed a fluorescence biosensor based on mercaptopropionic acid-functionalized CdTe QDs for the rapid detection of the breast cancer biomarker CA15-3 within 10 min. Li et al. [83] designed quantum dot-encoded magnetic microbeads based on microfluidic technology. The microbeads employ CdSe/ZnS core–shell quantum dots and Fe_3_O_4_ magnetic nanoparticles. Effective encapsulation via a Pickering structure constructed from silica nanoparticles avoids fluorescence quenching and toxicity issues. By designing a 4 × 4 barcoding system with two colors and four fluorescence intensities, they achieve high-throughput, ultra-sensitive detection of multiple tumor markers such as CEA, CA125, and CA19-9, exhibiting excellent detection performance. Wu et al. [84] developed an ultra-sensitive sensor combining fluorescence resonance energy transfer effects, utilizing green- and blue-emitting core–shell UCNPs as energy donors and MoS_2_ nanosheets as quenching media for the dual detection of miRNA-593 and miRNA-155.

While enzyme-linked immunosorbent assay remains the standard in biomedical research [85], it faces challenges in achieving highly sensitive, cost-effective, and efficient multiplexed detection. To address this, Wu et al. [86] developed a microfluidic fluorescence sensor integrated with nano-graphene oxide particles for the simultaneous detection of CA125, human epididymis protein 4 (HE4), CEA, and AFP. Liu et al. [87] proposed a fluorescence aptasensor based on endonuclease-mediated signal amplification, as shown in Figure 2C, utilizing magnetic nanoparticles to specifically identify CEA and AFP. Furthermore, technologies utilizing fluorescent RNA aptamers enable traceless detection of multiple analytes without external labeling [88]. Nucleic acid amplification techniques, such as rolling circle amplification (RCA) and hybridization chain reaction (HCR), are also frequently integrated; for instance, Guan et al. [89] combined rolling circle transcription with fluorescent RNA aptamers for the dual detection of AFP and CEA. This approach not only distinguished positive samples from healthy controls but also enabled field-deployable POCT. However, single-modal fluorescence sensing still faces systemic hurdles, including background interference from sample autofluorescence and the potential toxicity of certain fluorescent materials (e.g., cadmium-based QDs).

#### 3.2.3. SERS Sensors

SERS is a molecular spectroscopic technique that reveals vibrational and rotational information through the inelastic scattering of light [90,91,92]. Although traditional Raman scattering signals are too weak for sub-ppm level detection, SERS significantly amplifies these signals by utilizing molecules adsorbed on roughened metal surfaces or nanostructures. By combining the “fingerprinting” capability of Raman spectroscopy with the electromagnetic enhancement effects of nanostructured materials [93,94,95,96], SERS provides exceptional sensitivity and rich vibrational spectral information.

**Figure 3:**
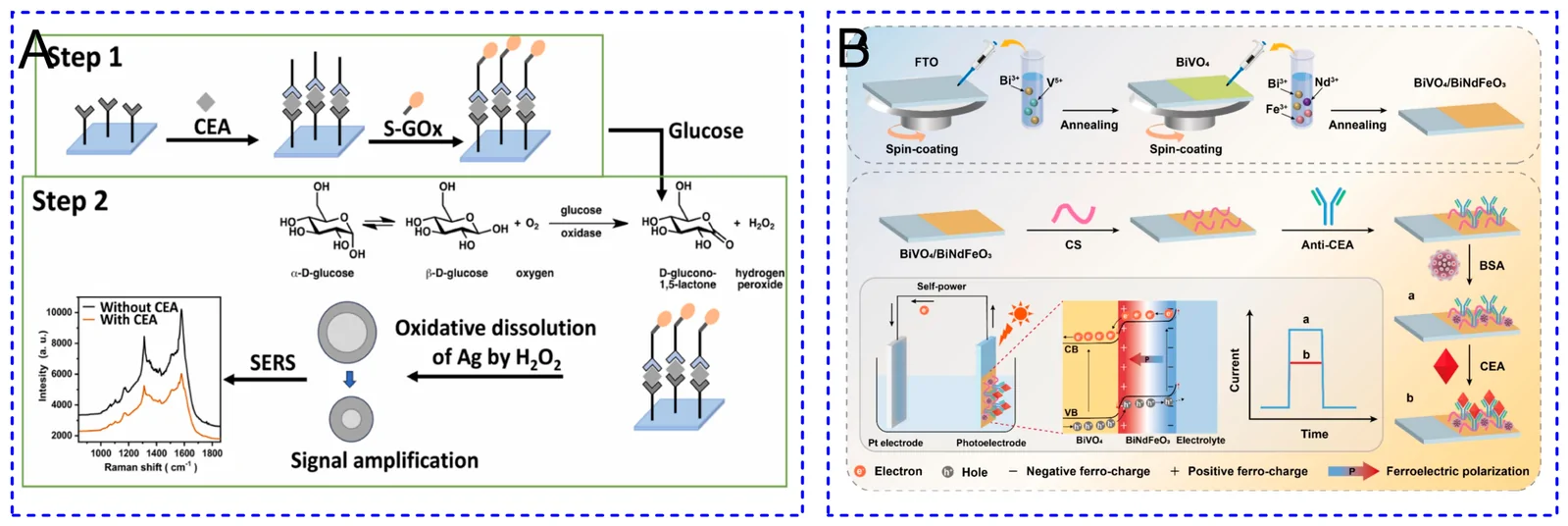
(**A**) Schematic mechanism of GOx-assisted SERS immunoassay and signal amplification. Reprinted with permission from ref. [99]. Copyright 2025, Alexandria Engineering Journal. (**B**) Schematic illustration of BVO/BNFO photoelectrode synthesis and mechanism of the self-powered PEC biosensor based on BVO/BNFO ferroelectric polarization for CEA detection. Reprinted with permission from ref. [118]. Copyright 2025, Chemical Engineering Journal.

The core of SERS tag design lies in engineering nanostructures with abundant “hotspots”. Nie et al. [97] and Yang et al. [98] developed Au@Ag core–shell structures and functionalized silver nanorod arrays, respectively, to establish high-density hotspots for ultra-sensitive biomarker capture. Geng et al. [99] prepared AgPb bimetallic core–shell nanoparticles (AgPb NPs) via a polyol reduction method; under GOx-catalyzed glucose oxidation, the silver shell was selectively oxidized, significantly enhancing the SERS signal (Figure 3A). Shao et al. [100] developed an aptasensor platform based on a one-step recognition-release mechanism using Au/Si nanoumbrella arrays, achieving fM-level detection of platelet-derived growth factor-B (PDGF-B) and thrombin. Similarly, Zhu et al. [101] leveraged specific aptamer recognition between Ag-Au core–shell nanorods (with Raman reporters) and SERS capture substrates to markedly enhance plasmonic coupling for ultra-sensitive detection.

With increasing demand for diagnostic accuracy, multiplexed detection has become paramount. Ouyang et al. [102] developed a sandwich-structured SERS sensor for the high-sensitivity quantification of chemokine ligand 20 and epidermal growth factor, significantly improving the efficiency of clinical therapeutic evaluation and offering new pathways for enhancing patient survival.

#### 3.2.4. PEC Sensors

PEC sensing is an emerging technology based on the light-induced excitation of photosensitive materials, which triggers the generation of charge carriers. It represents a synergistic process of photoexcitation and electrochemical detection [103,104]. By combining electrical and photochemical techniques, PEC sensors maintain a high signal-to-noise ratio and excellent reliability even at low analyte concentrations [105]. Unlike traditional electrochemical methods, PEC achieves complete separation between the excitation source (light) and the detection signal (electricity), thereby partially suppressing background interference [106,107].

The development of novel photosensitive materials and signal amplification strategies has further propelled PEC performance. However, overcoming low conductivity and rapid charge carrier recombination remains a significant challenge. Zong et al. [108] utilized catalytic hairpin assembly for signal amplification, enhancing photocurrent through the exciton-plasma coupling between AuNPs and CdTe QDs for the specific detection of AFP and miRNA-122. Naz et al. [109] addressed recombination issues by applying plasmonic Au NPs to Co_3_O_4_ nanostructures, enabling sensitive detection of folate-binding protein (FBP).

To optimize photoelectric conversion efficiency, the construction of heterojunctions is a central strategy [110]. Active materials (e.g., TiO_2_, ZnO, CdS) [111,112,113] generate photogenerated electron-hole pairs under illumination, which are sensed as photocurrent in response to the target analyte. Li et al. [114] constructed a BiOI/CdS/TiO_2_ Z-scheme heterojunction that effectively promoted the separation of photogenerated electron-hole pairs, vastly improving the photocurrent response for AFP detection. Sakib et al. [115] utilized Ag NP-modified TiO_2_ nanoparticles to achieve enhanced photocurrent for microRNA sensing. Furthermore, the built-in electric field formed at the heterojunction interface provides an electrostatic force for regulating charge transfer [116]. Cheng et al. [117] combined Bi_2_S_3_/Bi_2_O_2_(OH)_2_ nanorods with Mo_2_C:N@Ru nanospheres to develop a “signal-off” sensor for CYFRA21-1. Notably, self-powered PEC platforms, which operate without external power, have simplified device architectures. Liu et al. [118] utilized the polarization effect of ferroelectric materials to construct a self-powered platform for CEA detection (Figure 3B). Nevertheless, the long-term stability of photoelectrodes in biological fluids remains a challenge, and single-modal photocurrent signals struggle to distinguish between signal decreases caused by target binding versus those caused by electrode corrosion or delamination. Additionally, the environmental and biological compatibility of heavy-metal-containing photosensitizers warrants consideration.

Although colorimetry, fluorescence, SERS, and PEC are the main optical modalities discussed in this review, it is worth noting that there is also a complementary group of label-free quantitative optical methods that extract quantitative information from the intrinsic optical properties of cells and extracellular vesicles (EVs). Digital holographic microscopy (DHM) records the phase delay of light after it passes through a sample, allowing label-free reconstruction of cell morphology and dry mass distribution. Wittenzellner et al. [119] combined DHM with machine learning to perform single-cell phenotyping of pancreatic ductal adenocarcinoma cells, tracking dynamic changes in tumor cell differentiation and heterogeneity in real time. For EV analysis, digital holographic tomography (DHT) extends DHM into three dimensions. The DISEP kinetic assay developed by Zhai et al. [120] uses low-affinity aptamer probes to transiently capture individual tumor-derived EVs and track their binding kinetics in real time, achieving an AUC of 0.98 in distinguishing prostate cancer patients from healthy donors based on EV surface protein signatures. Interferometric scattering microscopy (iSCAT) follows a similar idea, estimating EV size and refractive index from interferometric contrast signals. Recent work has combined iSCAT with nanotweezers and artificial intelligence for rapid, parallel capture and label-free analysis of EVs [121]. These methods offer a complementary route to multimodal sensing that differs from fluorescence and electrochemistry. The physical quantities they provide—phase, refractive index, and size—are complementary in information dimension to biochemical signals, and integrating them with existing multimodal platforms may provide richer multidimensional data for tumor biomarker detection.

### 3.3. Section Summary

In summary, while electrochemistry provides high sensitivity, colorimetry offers intuitive readouts, and SERS delivers molecular fingerprinting, single-modal biosensors each hold distinct value for specific applications. However, they share a systemic vulnerability: relying on a single signal source makes detection highly susceptible to matrix interference, environmental fluctuations, and operational errors, leading to persistently high false-positive or false-negative rates. This “single-signal” dependence lacks internal calibration, making it difficult to self-validate anomalous signals. Consequently, meeting the rigorous “high-reliability” standards of precision medicine requires breaking down the barriers of single-modal mechanisms. Integrating two or more complementary sensing modes onto a single platform to build dual-modal and multi-modal biosensors with cross-validation capability is now essential for overcoming current detection bottlenecks and precisely identifying tumor markers. Fundamentally, this transition marks a paradigm shift from single-source reliance to multi-signal collaborative validation. By incorporating orthogonal physicochemical transduction mechanisms, these distinct signals corroborate one another, effectively distinguishing genuine target responses from matrix interference, environmental fluctuations, and operational errors. Building on this, multifunctional nanomaterials and ratiometric signal designs further broaden the dynamic range and enhance both sensitivity and anti-interference capability. Ultimately, as modalities expand from dual-modal to multi-modal and integrate with machine learning for high-dimensional data fusion, combinatorial sensing strategies are advancing from simple signal superposition toward intelligent, multidimensional precision diagnostics—offering a systematic pathway for reliable tumor biomarker detection in complex clinical settings.

## 4. Electrochemical Sensing-Integrated Dual-Modal Sensing Strategies

Dual-modal strategies integrate two orthogonal or complementary signal transduction mechanisms within a single sensing interface, leveraging diverse physicochemical principles for the synchronous response to a target analyte [122]. This approach not only effectively suppresses environmental background noise via self-calibration mechanisms but also integrates the unique merits of different modalities (e.g., high sensitivity and easy operability), greatly improving the accuracy of analytical outputs. True cross-validation can only be achieved when the two sensing modalities are based on different physicochemical signal-transduction principles and their signal generation processes remain mutually independent. Such a research paradigm has significantly propelled the development of biosensing technology and has emerged as one of the most prominent frontiers in disease diagnostics.

### 4.1. Electrochemical-Colorimetric Dual-Modal Sensors

In the application of electrochemical-optical dual-modal platforms, the electrochemical-colorimetric (EC-CL) approach is currently the most widely adopted dual-modal strategy. In the early 1990s, Hapiot et al. utilized various electrochemical methods and pulse radiolysis to investigate the oxidation of syringaldazine in acetonitrile and aqueous media; the reversible two-electron/two-proton transfer resulted in the formation of a deep purple compound directly observable by the naked eye [2]. Electrochemistry provides high-sensitivity quantitative analysis, while colorimetry offers intuitive “naked-eye” qualitative assessment. The synergistic combination of these characteristics has since propelled EC-CL sensing into the scientific mainstream.

**Figure 4:**
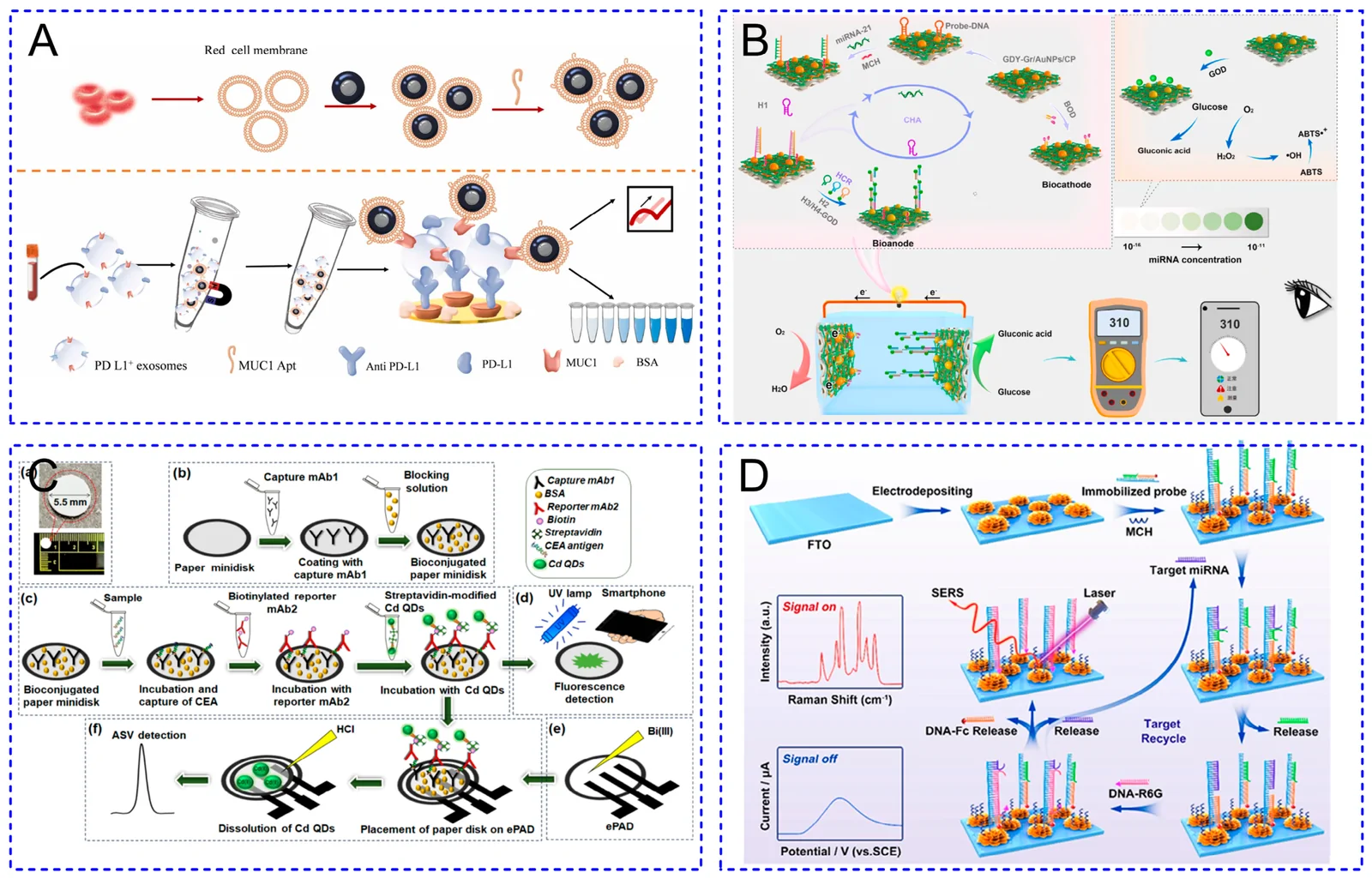
(**A**) Schematic of the PD-L1^+^ exosome sensing strategy utilizing dual-signal magnetic-plasmonic nanocomposites for signal amplification. Reprinted with permission from ref. [124]. Copyright 2025, Sensors and Actuators B: Chemical. (**B**) Construction of a dual-modal self-powered sensing platform. Reprinted with permission from ref. [127]. Copyright 2024, Sensors and Actuators B: Chemical. (**C**) (**a**) Photograph and dimensions of the diagnostic paper mini-disc; (**b**) Bioconjugation of the disc with capture mAb1; (**c**) Immunoassay; (**d**) Fluorescence detection of CEA; (**e**) Modification of ePAD with Bi (III); (**f**) Electrochemical detection of CEA. Reprinted with permission from ref. [132]. Copyright 2020, Analytica Chimica Acta. (**D**) Schematic strategy for enzyme-free target recycling amplification detection of miRNA. Reprinted with permission from ref. [141]. Copyright 2021, Analytical Chemistry.

Significant breakthroughs in nanotechnology have opened new avenues for colorimetric analysis. The utilization of nanomaterials with peroxidase-like mimicking activity (e.g., MOFs and nanozymes) is critical for constructing EC-CL detection platforms. For instance, Tang et al. [123] employed Fe-MOF both as an electrochemically active probe and as a high-efficiency catalyst for 3,3’,5,5’-tetramethylbenzidine (TMB) chromogenesis, achieving dual confirmation of liver cancer cells through “electrical signals + naked-eye observation”. This work also underscores the vast potential of integrated microfluidic chips in the precise, early point-of-care diagnosis of hepatocellular carcinoma. Liu et al. [124] proposed an EC-CL strategy based on silver nanoparticle-decorated magnetic nanoparticles (Ag@MNPs). Leveraging the magnetism, electrochemical activity, and peroxidase-like activity of Ag@MNPs, they achieved dual-target recognition and high-sensitivity detection of programmed cell death ligand 1 (PD-L1) and MUC1 on exosome surfaces (Figure 4A). Its exceptional analytical performance enables the accurate processing of complex serum samples, effectively distinguishing healthy individuals from cancer patients. Gong et al. [125] utilized molybdenum disulfide nanozymes modified with Au@Pt core–shell nanoparticles to catalyze the TMB-H_2_O_2_ reaction, generating signals for detecting exosomes from the breast cancer cell line MCF-7. This dual-modal aptasensor provides an innovative solution for developing high-precision biomolecular detection platforms.

To circumvent the requirement for external power sources, Shi et al. [126] developed a smartphone-based self-powered EC-CL platform. By integrating binary 3D DNA walkers, the CRISPR/Cas12a system, and ultrathin two-dimensional (2D) graphdiyne materials, they achieved ultra-sensitive detection of the breast cancer biomarker miRNA let-7a with a LOD as low as 8.11 aM, offering a reliable solution for remote monitoring and field-based testing. Xu et al. [127] constructed a self-powered sensing platform based on graphdiyne heterojunctions, enabling dual detection of miRNA-21 via smartphone readouts (Figure 4B). This significantly lowers the technological threshold for field analysis and future mobile health services. The core advantage of the EC-CL dual-mode lies in its “dual-assurance” mechanism: colorimetry provides rapid “positive/negative” initial screening to prevent misinterpretation caused by electrochemical signal drift, while electrochemistry provides precise concentration values when colorimetric sensitivity is insufficient. This modality is particularly suited for cancer screening in resource-limited settings.

The main strength of EC-CL lies in combining high-sensitivity quantification with intuitive visual readouts, but its key limitation is the susceptibility of the colorimetric signal to sample coloration—hemolyzed, icteric, or lipemic samples can severely interfere with colorimetric readings, while the electrochemical signal may suffer from baseline drift due to electrode surface fouling. To mitigate this, recent studies have adopted dual-wavelength ratiometric measurements or introduced internal reference signals to correct background absorption, along with antifouling electrode coatings to reduce matrix adsorption. For validation, this strategy should at least include standard addition recovery tests in complex biological matrices and comparison with clinical reference methods such as ELISA or chemiluminescence to confirm consistency between the two modes.

### 4.2. Electrochemical-Fluorescence (EC-FL) Dual-Modal Sensors

Unlike colorimetry, fluorescence methodologies inherently possess exceptional sensitivity. In the mid-1980s, Howells et al. [128] utilized an electrochemistry-fluorescence (EC-FL) dual mode for the detection of pterins, dihydropterins, and tetrahydropterins, which stands among the earliest implementations of such dual-modality strategies for biomarker analysis. Compared to single-modal approaches, the EC-FL dual-mode may aid in improving diagnostic accuracy.

In recent years, the EC-FL modality has emerged as a premier research avenue in oncology. Multifunctional nanomaterials, with their superior optical and electrochemical properties, not only markedly enhance fluorescence sensitivity but also simplify sensor design to achieve efficient multi-signal output. Korram et al. [129] synthesized blue-emitting S-doped carbon dots with a size of about 3 nm via hydrothermal treatment of onion extract and bioconjugated them with horseradish peroxidase-labeled prostate-specific antigen (PSA) antibodies for precise PSA detection. This platform was further integrated into paper-based and smartphone-integrated fluorescence immunoarrays, offering a rapid and cost-effective detection means. Zhao et al. [130] combined magnetic Fe_3_O_4_ nanoparticles with CEA antibodies to form polyamide complexes, utilizing carbon nanotubes and carboxylated CdSe nanocrystals as a signal amplification system for high-performance EC-FL response detection of CEA. Zhang et al. [131] utilized ZIF-67@Sb nanocomposites to capture human epidermal growth factor receptor 2 (HER2) proteins, simultaneously triggering the release of methylene blue (MB) and TMB for synchronous EC-FL signal “turn-on,” enabling rapid and high-precision detection of dual targets in tissue and serum samples within 30 min. In EC-FL detection, “signal switching” strategies (turn-on/turn-off) are often designed to mitigate background interference.

Quantum dots (QDs) are ideal candidates for EC-FL dual-modal detection due to their dual optoelectronic characteristics. Mermiga et al. [132] utilized Cd QDs both as fluorescent emitters and as sources for Cd^2+^ ions (via acid digestion) for anodic stripping voltammetric detection, achieving ultra-sensitive analysis of CEA. This “homologous-source/divergent-signal” design ensures the consistency of dual-modal signals from the material origin (Figure 4C). Li et al. [133] developed MoS_2_/ferrocene/Pd nanoparticles (MoS_2_-Fc-PdNPs) and nitrogen-doped graphene QDs for detecting serum Golgi protein 73 (GP73) concentrations. In this system, MoS_2_-Fc-PdNPs act as fluorescence quenchers and concurrently amplify electrochemical signals.

The core challenge for EC-FL is “signal cross-talk”: potential changes during electrochemical scanning may quench or photobleach fluorescent probes, while fluorescence excitation light can also perturb the electrochemical background current. To reduce such interference, researchers have developed “signal-switching” strategies and orthogonal time-resolved acquisition modes, so that the two signals are generated in separate time windows or potential ranges. In addition, autofluorescence and light scattering, which are inherent to fluorescence detection, are particularly problematic in complex biological samples. Validation should therefore focus on whether the signal-to-noise ratio and detection limit of the fluorescence channel are maintained in real matrices. At a minimum, validation should include recovery tests in serum or plasma (typically 80–120%), intra- and inter-assay precision (RSD preferably below 15%), and consistency analysis against conventional methods. Mermiga et al. [132] used Cd QDs as both fluorescent emitters and electrochemical detection sources for dual-modal CEA detection; their “same-source/divergent-signal” design ensured material consistency between the two modes and offers a useful reference for validation strategies. The integration of electrochemical and fluorescent techniques goes beyond mere superposition of individual functions. Instead, these dual-modal platforms pursue ultralow detection limits and leverage ratiometric readouts to correct background environmental interferences.

### 4.3. Electrochemical-SERS (EC-SERS) Dual-Modal Sensors

SERS provides profound molecular structural information via its distinctive “fingerprinting” capability. Its intense Raman scattering signals enable effective discrimination between complex analytes with exceptional specificity [134], rendering it a robust tool for both qualitative and quantitative diagnostics [135,136]. Conversely, EC methods are optimized for rapid quantification. The EC-SERS sensing platform, formed by integrating these two detection modalities, is regarded as an optimal strategy to resolve false-positive signals in complex clinical samples. While the integration of Raman spectroscopy with electrochemistry dates back to the 1970s, its utilization in dual-modal disease diagnostics has flourished since the early 21st century.

Recently, a diverse array of functional nanomaterials has been developed to modify electrode surfaces to enhance electrochemical sensing. Specifically, metal nanoparticles with engineered cores (e.g., Au NPs, Ag NPs) exhibit excellent biocompatibility and serve as powerful amplifiers for SERS signals. Castano-Guerrero et al. [137] immobilized antibodies on a cysteamine-modified Au-SPE to establish an EC sensing interface, followed by the introduction of gold nanostar-labeled secondary antibodies for SERS analysis. This complementary EC-Raman signal output paves the way for multiplexed detection and large-scale applications. Luo et al. [138] developed a novel SERS/EC dual-modal nanosensing platform based on Au@Ag nanowires for miRNA-141 detection, successfully discriminating the target from interfering miRNAs. He et al. [139] constructed a DNA hybridization system on gold nanoparticle-modified electrodes and immobilized peroxidase-mimicking Mn_3_O_4_/S4 complexes. In the presence of the target, an enzyme-assisted recycling reaction triggered dual EC-SERS signal output. This innovative signal conversion mechanism offers new perspectives for dual-modal nucleic acid detection on a single interface.

The structural morphology of SERS substrates exerts a profound influence on signal intensity, prompting exploration of complex metallic architectures. 3D nanostructures—such as star-like, flower-like, dendritic, or urchin-like morphologies-demonstrate superior SERS enhancement due to the abundance of plasmonic “hotspots” [140]. Zhou et al. [141] designed a “popcorn-like” gold nanofilm that simultaneously provides dense SERS hotspots and a significantly increased electrochemically active surface area. This structural synergy enabled synchronous amplification of EC-SERS signals for miRNA-21 detection. Such modular designs can be extended to various cancer-related nucleic acid biomarkers by substituting the probe sequences (Figure 4D). Gu et al. [142] utilized electrodeposition to fabricate a charge-selective gold microelectrode array, combined with hybridization chain reaction amplification, to construct a “super-sandwich” architecture for the high-sensitivity multiplexed detection of AFP and CEA.

**Figure 5:**
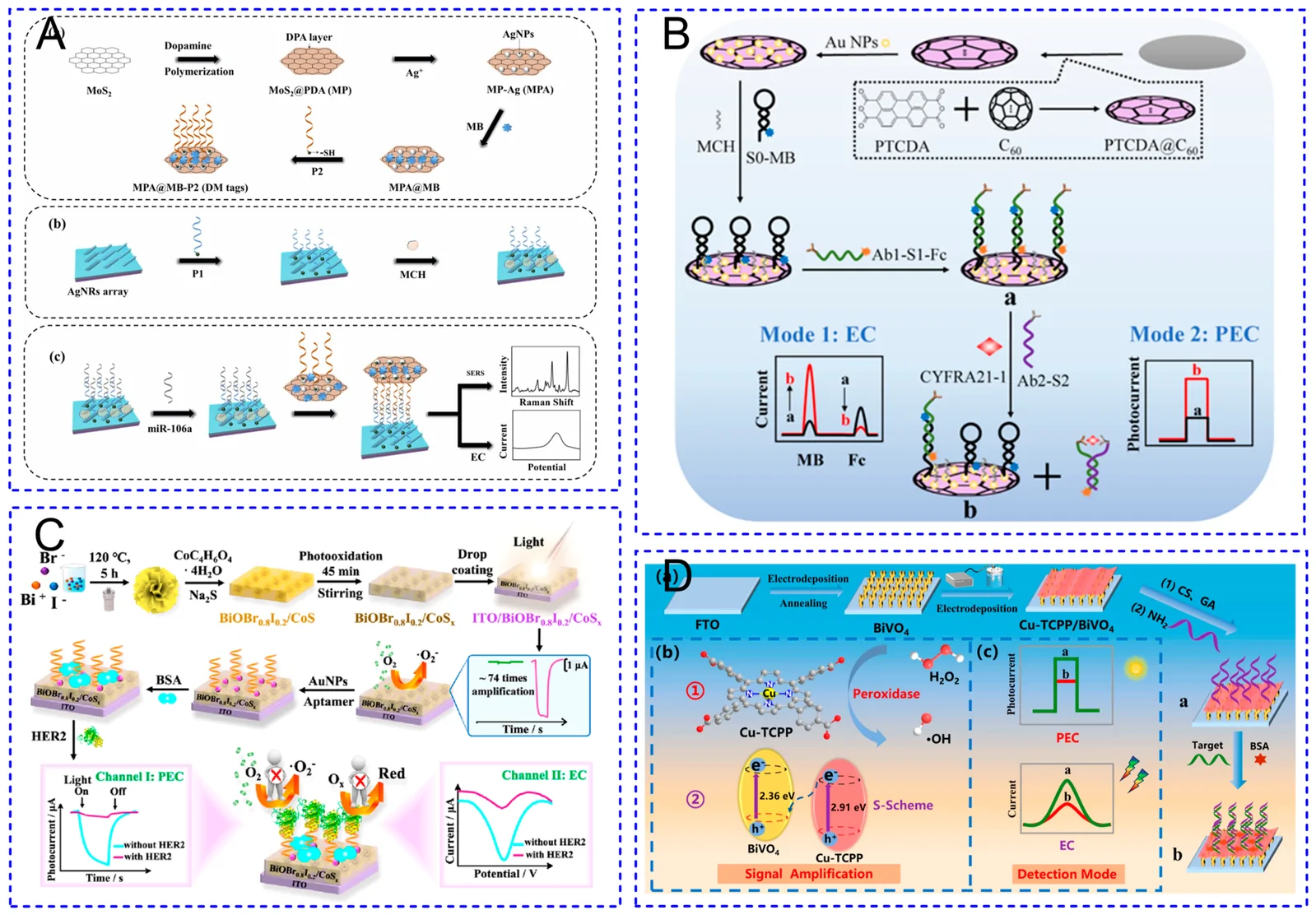
(**A**) Schematic illustration of the dual-modal sensing system for miR-106a based on DM tags and AgNRs. Reprinted with permission from ref. [22]. Copyright 2022, Sensors and Actuators B: Chemical. (**B**) Schematic of the dual-modal sensing platform for CYFRA21-1 detection. Reprinted with permission from ref. [20]. Copyright 2021, Analytical Chemistry. (**C**) Schematic of the HER2 dual-modal platform based on BiOBr_0.8_I_0.2_/CoSx p−n heterojunctions. Reprinted with permission from ref. [152]. Copyright 2022, ACS Applied Nano Materials. (**D**) (**a**) Construction process of the biosensor. (**b**) Schematic of light-nanozyme signal amplification. (**c**) Mechanism of dual-modal sensing. Reprinted with permission from ref. [154]. Copyright 2025, Analytical Chemistry.

2D materials, such as MoS_2_ and WS_2_, enhance electrode reactivity through their exceptional conductivity and anti-interference properties. When combined with Au nanoparticles, the synergy between electromagnetic and chemical enhancement significantly boosts SERS intensity [143]. Yaiwong et al. [144] utilized Au nanoparticle-modified molybdenum disulfide nanocomposites (Au@MoS_2_/SPE) in a sandwich immunoassay to form EC-SERS dual-responsive nanotags for accurate PSA quantification in human serum. Zhai et al. [22] developed a sensor based on MoS_2_ nanosheets and Ag nanorod arrays (Figure 5A) to discriminate the gastric cancer biomarker miRNA-106a from its single-base mismatched sequences. The molecular fingerprinting capability of SERS was pivotal here, confirming the reliability of the sensor in complex biological matrices. The fusion of SERS and electrochemical sensing represents a unique value proposition for high-precision diagnostic analysis.

The combination of EC and SERS offers the unique advantage of “quantitative analysis plus molecular fingerprinting,” but batch-to-batch reproducibility of SERS substrates remains its most prominent shortcoming. Variations in the size, shape, and hotspot distribution of metal nanoparticles can cause significant fluctuations in SERS signal intensity, while reaction byproducts generated during electrochemical potential scanning may contaminate or damage the plasmonic coating and compromise signal stability. To mitigate this, researchers have used templated nanofabrication to ensure uniform hotspot distribution or introduced internal standard molecules such as rhodamine 6G to normalize SERS signals. For validation, EC-SERS platforms should focus on evaluating the coefficient of variation between substrates (a target value below 15% is recommended) and verify reproducibility across multiple independently prepared substrates. In addition, since SERS provides structural fingerprint information, validation should also examine its selectivity in distinguishing structurally similar analogues, ensuring that the specificity of the dual-modal signal is not supported solely by the fingerprinting capability of SERS.

### 4.4. Electrochemical-Photoelectrochemical (EC-PEC) Dual-Modal Sensors

EC-PEC dual-modal sensors integrate photocurrent detection and electrochemical techniques under optical stimulation. By utilizing the same detection unit and instrumentation, dual signals can be acquired synchronously, often employing a “ratiometric measurement” strategy where the ratio of photocurrent to dark current cancels out systemic errors [145,146]. Compared to dual-modal platforms requiring multiple instruments, this strategy streamlines operational protocols and markedly enhances applicability and accuracy in real-sample analysis.

Ratiometric electrochemical strategies that simultaneously read out “signal-on” and “signal-off” states have garnered significant attention due to their intrinsic stability and reproducibility [147]. Through their self-referencing capability, these strategies effectively mitigate signal fluctuations caused by environmental perturbations [148]. Zhang et al. [20] proposed an “on-off-on” ratiometric strategy utilizing the divergent directions of photocurrent and electrochemical current changes (Figure 5B). This built-in calibration curve successfully compensated for systemic errors caused by probe delamination or environmental variations, substantially improving the reproducibility of CYFRA 21-1 detection in serum.

The design of photosensitive materials is critical to PEC performance [149,150]. Wu et al. [151] prepared BiVO_4_/BiOI-MWCNT photosensitive materials on Fluorine-doped Tin Oxide glass and functionalized the photoelectrode for label-free detection of AFP. Wang et al. [152] synthesized a BiOBr_0.8_I_0.2_/CoSx nanocomposite with high PEC and EC activities via a hydrothermal method. Upon conjugation with HER2 aptamers, in situ visible light irradiation significantly enhanced the photocurrent for ultra-sensitive HER2 detection (Figure 5C). Wei et al. [153] constructed a Au NPs/CdS NS heterostructured platform. The SPR effect of Au NPs significantly amplifies both PEC and EC signals under illumination, enabling dual-modal detection of HE4. Li et al. [154] designed an S-scheme heterojunction based on Cu-TCPP and BiVO_4_ for miRNA-133a sensing. This architecture not only promoted the separation of photogenerated carriers but also exhibited enzyme-like activity to catalyze electrochemical reactions (Figure 5D). This “two-pronged” approach at the material level represents a paradigm of dual-modal sensing design, showcasing immense potential for early disease diagnosis.

The main advantage of EC-PEC is the natural separation between the excitation source (light) and the detection signal (electricity), which gives it a lower background than conventional electrochemical methods. However, the long-term stability of photoelectrodes in biological fluids remains a major bottleneck—photoactive nanomaterials such as metal oxides and quantum dots may undergo photocorrosion or decreased charge separation efficiency under continuous illumination and electrochemical bias, leading to photocurrent decay over time. To address this limitation, ratiometric measurement strategies have been developed, using the ratio of photocurrent to dark current as the output signal to compensate for systematic errors caused by electrode delamination and environmental fluctuations. In addition, reference-electrode-based closed bipolar electrode structures have been shown to provide a stable potential reference, enhancing the photocurrent responses by up to 40-fold. For validation, EC-PEC platforms should pay particular attention to the signal decay rate of photoelectrodes under continuous illumination, ideally monitoring photocurrent response periodically over at least 10 days to assess long-term stability, while also using standard addition methods in clinical samples to confirm consistency between the two modes. For example, a PEC-EC dual-modal sensor for miRNA-133a achieved detection limits of 0.003 fM and 0.02 fM for the PEC and EC modes [154], respectively, with good selectivity and reproducibility in real samples, providing a useful reference for validation criteria.

### 4.5. Section Summary

The integration of EC and optical sensing modalities has emerged as a cornerstone of state-of-the-art dual-signal bioanalysis. By leveraging advanced signal conversion and sensing mechanisms, these dual-modal platforms enable both intuitive observation and high-precision quantification of biomarkers. This convergence not only streamlines experimental workflows but also reduces reliance on bulky, specialized analytical equipment. Incorporating multifunctional nanomaterials and innovative sensing strategies into unified EC-optical systems has significantly improved detection performance and operational stability. This fusion of technologies continues to catalyze innovation, providing robust solutions for diverse clinical and bioanalytical challenges.

## 5. Multi-Modal Sensing

Currently, while dual-modal systems are a focal point of contemporary research, there is an increasing shift toward the development of multi-modal sensing platforms capable of generating three or more distinct signals. This paradigm shift aims to further revolutionize sensor functionality by merging three or more sensing elements or transduction mechanisms. Such multi-modal systems can synchronously or sequentially measure analytes through diverse signal modalities, including electrical, optical, chemical, thermal, and mechanical forms [155]. When the signal pathways are sufficiently independent, these systems may enhance diagnostic accuracy through cross-validation; otherwise, they may share common error sources. Consequently, the advancement of multi-modal sensing architectures is of paramount importance across various scientific disciplines.

**Figure 6:**
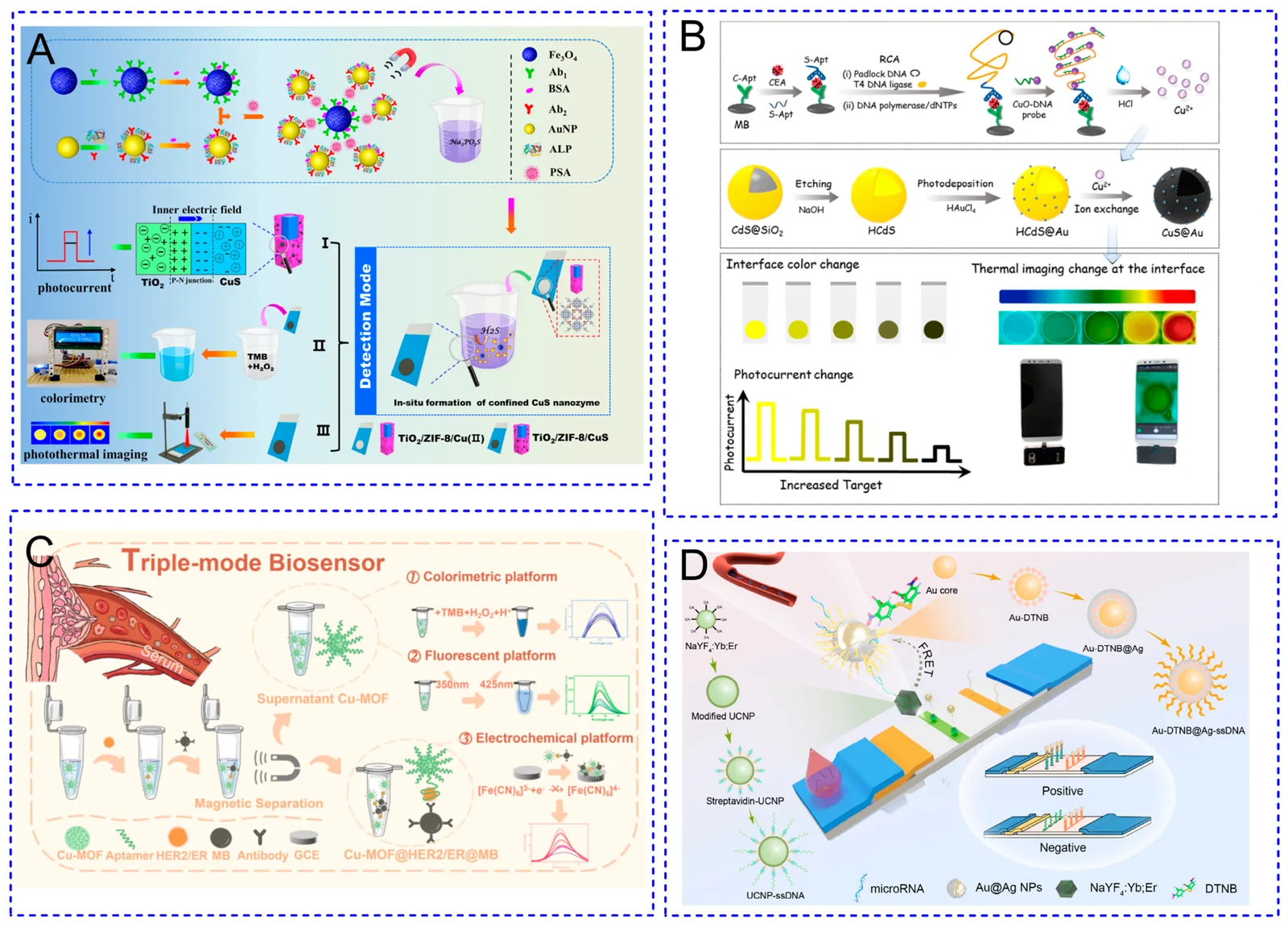
(**A**) Multi-modal biosensor based on TiO_2_/ZIF-8/Cu(II). Reprinted with permission from ref. [156]. Copyright 2024, Analytical Chemistry. (**B**) Schematic of an integrated PEC-Visual multi-modal biosensor based on 3D hollow HCdS@Au nanospheres coupled with RCA and target-induced ion exchange. Reprinted with permission from ref. [157]. Copyright 2022, Analytical Chemistry. (**C**) Schematic of trimodal HER2/ER detection. Reprinted with permission from ref. [158]. Copyright 2024, ACS Applied Nano Materials. (**D**) Schematic and synthetic route for a multi-modal lateral flow biosensor for miRNA detection. Reprinted with permission from ref. [159]. Copyright 2024, Biosensors & Bioelectronics.

Multi-modal detection strategies can be broadly categorized into parallel and sequential approaches. Parallel strategies facilitate the simultaneous acquisition of information through multiple channels. For example, Meng et al. [156] developed an innovative trimodal parallel sensing platform (PEC-Colorimetric-Photothermal imaging). By employing an in situ MOF engineering strategy, they successfully fabricated a multifunctional integrated TiO_2_/ZIF-8/Cu(II) electrode (Figure 6A), which effectively addressed the persistent challenges of high false-positive/negative rates in single-modal PSA detection. Sequential strategies, on the other hand, utilize external stimuli, such as acidolysis or illumination, to trigger distinct signals stepwise for target detection. Zhou et al. [157] constructed a multi-modal platform based on 3D hollow HCdS@Au nanospheres for the sequential detection of CEA via PEC, colorimetry, and photothermal imaging (Figure 6B). Following RCA, the acidolysis of CuO-DNA probes releases Cu^2+^ ions, which undergo ion exchange with the electrode material to generate a dark Cu nanostructure. This results in a simultaneous decrease in photocurrent and a visual color transition, while enabling photothermal visualization via an infrared camera.

Multi-modal biosensors enhance analytical accuracy, analyte coverage, and comprehensive detection capability by integrating complementary sensing technologies. Liu et al. [158] developed a trimodal nanosensor based on the synergy between a Cu-MOF and magnetic beads for integrated electrochemical, colorimetric, and fluorescence detection (Figure 6C). This innovative approach utilized the Cu-MOF in the supernatant as the colorimetric and fluorescent sensing component, while the Cu-MOF@antigen@MB complex served as the electrochemical transducer, enabling high-sensitivity parallel detection of the breast cancer biomarkers HER2 and estrogen receptor (ER). Similarly, Chen et al. [159] developed a trimodal lateral flow assay system utilizing upconversion nanoparticles for colorimetric, fluorescent, and Raman sensing of miRNA-21 (Figure 6D). In practical clinical applications, this system accurately discriminated patients with lung cancer and periodontitis from healthy cohorts.

The studies discussed above have already demonstrated the feasibility of multimodal sensing for protein and nucleic acid markers. Recent work has pushed this idea further toward more complex clinical samples and a wider range of marker types. For particle- and cell-level markers such as tumor-derived vesicles and circulating tumor cells (CTCs), Zhang et al. [160] combined hierarchical FeZr-MOF@CuFe-LDH heterostructure nanozymes with dual-aptamer sandwich recognition and magnetic separation to construct a chemiluminescence/electrochemical/colorimetric (CL/EC/CM) trimodal aptasensor for detecting lung cancer-derived extracellular vesicles (tEVs). The platform simultaneously recognized CD63 and EGFR on tEV surfaces and showed good discrimination between serum samples from lung cancer patients and healthy donors. Lin et al. [161] developed a trimodal lateral flow assay platform based on Au@PtOs nanoclusters, in which a single nanolabel simultaneously generated catalytic colorimetric, SERS, and photothermal signals for multimodal detection of breast cancer exosomes. The SERS mode further enabled different breast cancer exosome subtypes to be distinguished through their spectral fingerprints. Following a similar integration strategy, Wang et al. [162] integrated antifouling magnetic nanoparticles, dual-targeting recognition, and hybridization chain reaction into a single system; using G-quadruplex/hemin DNAzyme to catalyze ABTS oxidation, they built an absorbance/colorimetric/photothermal trimodal sensing platform for circulating tumor cell detection. With a three-channel signal array and machine learning, the method distinguished five types of CTCs and achieved an AUC of 0.965 in serum samples from cervical cancer patients. Both studies placed recognition, separation, and signal amplification in a single workflow. The main difference lies in the readout: the former uses CL/EC/CM to cross-validate dual protein markers on EVs, while the latter uses absorbance/colorimetric/photothermal signals combined with machine learning to distinguish CTCs from different sources. Building upon these findings, Debbarma et al. [163] pushed multichannel detection to the level of intact cells by designing a four-channel photonic crystal fiber SPR sensor capable of detecting four cancer cell types—blood, breast, cervical, and adrenal—in a single test. This illustrates the promise of parallel multichannel sensing for simultaneous multi-cancer screening.

Turning from cells and vesicles to small-molecule metabolites and protein markers, Wang et al. [164] combined CeO2-TPE fluorescent nanozymes with smartphone readout to design a fluorescence/colorimetry/smartphone RGB trimodal biosensor. In this system, SOX catalyzes the conversion of sarcosine to H_2_O_2_, which then regulates TMB oxidation and the fluorescence inner filter effect, enabling simultaneous detection of sarcosine and PSA in male body fluids. This shows that multimodal readout is not limited to nucleic acids or cells; with a well-designed signal transduction pathway, small molecules and protein markers can also be incorporated into the same detection logic. For nucleic acid markers, combining CRISPR/Cas12a with isothermal amplification offers another multimodal route. Dong et al. [165] proposed a CRISPR/Cas12a-powered trimodal sensor, CPTMB, in which R-LAMP amplification activates Cas12a. Activated Cas12a cleaves the hairpin biogate on MB@Fe-MOF and releases methylene blue, generating electrochemical/fluorescence/UV-vis signal outputs for miRNA-155 detection; the results agreed with RT-qPCR. Building on these results, Zhang et al. [166] further adopted a dual-CRISPR/Cas12a design, coupling EXPAR, DSN, and HCR with colorimetric, fluorescence, and electrochemical readouts for miRNA let-7a detection. The three modes showed good cross-validation and were validated in serum and cell samples. Overall, these studies extend multimodal sensing from single-marker detection to the combined analysis of EVs, CTCs, miRNAs, and small-molecule/protein markers. They also suggest that multimodal readout in complex clinical samples is gradually moving from simple signal stacking to mutual verification.

By integrating multiple signal outputs, these multi-modal sensors that consist of three or more detection modes deliver a more comprehensive evaluation of tumor biomarker levels and lay a solid foundation for clinical decision-making. These platforms exploit the complementary merits of various sensing technologies. For diagnostic accuracy, cross-validation can partially offset the limitations of single detection methods. For analytical capacity, distinct detection ranges work synergistically to expand the dimensionality of comprehensive biomarker characterization. This enables both the simultaneous detection of multiple targets and the acquisition of more dynamic and complete biochemical profiles through temporal monitoring. However, this technological convergence introduces significant engineering challenges, including increased architectural complexity, potential signal interference and crosstalk, and the necessity for more sophisticated calibration frameworks to ensure consistency and comparability across multi-source data. Navigating the trade-off between performance enhancement and system complexity remains the pivotal frontier in the development of next-generation multi-modal sensors.

## 6. Comprehensive Performance Comparison

Single-modal sensors rely on a single physical or chemical mechanism for detection, with their primary advantages centered on simplicity and cost-effectiveness. However, they exhibit significant inherent limitations, including systemic flaws within the sensing mechanism itself (e.g., interfacial interference, spectral overlap) and perturbations arising from the complexity of biological specimens (e.g., matrix effects and cross-reactivity). In contrast, dual-modal and multi-modal sensors derive their primary competitiveness from integrated performance enhancements. The fusion of multiple sensing modes is of profound significance across diverse research fields.

### 6.1. Single-Modal Sensors

Single-modal sensors remain the preferred tool for preliminary screening due to their straightforward design and implementation. Nevertheless, as previously delineated, their “single-signal” dependency makes them inadequate when dealing with complex biological specimens. They lack the capability for inter-molecular structural or state cross-validation, thereby limiting their ability to minimize misdiagnosis in precision medicine. Under conditions involving noise, drift, or interference, the accuracy of single-modal sensors is substantially compromised due to weak anti-interference capabilities. For example, during long-term operation of electrochemical sensors, electrode surface fouling or oxidation can increase interfacial resistance and reduce electron transfer efficiency, leading to signal attenuation or drift. In addition, biological matrices contain abundant macromolecular species (e.g., proteins and cellular debris) that spontaneously adsorb onto electrode interfaces; this process modifies interfacial properties and leads to severe resistance drift in EIS measurements [167]. In multiplexed biomarker detection, single-modal sensors—such as fluorescence platforms—frequently suffer from selectivity degradation due to overlapping emission spectra.

### 6.2. Dual-Modal and Multi-Modal Sensors

Two or more signals originating from the same recognition event (such as the same probe, the same nanomaterial, or the same amplification pathway) do not necessarily provide independent cross-validation or self-calibration, because they share the same error sources. For instance, if both the electrochemical and fluorescence signals arise from the redox reaction and fluorescence quenching of the same nanomaterial, then contamination of the electrode surface or degradation of the nanomaterial will affect both signals simultaneously. By increasing the number of information dimensions, dual-modal and multi-modal integration can partially overcome the accuracy limitations inherent in single-modal mechanisms when the integrated signals are sufficiently independent. For example, ratiometric signal outputs of dual-modal platforms can help reduce systematic errors arising from environmental perturbations when the two signals are independently affected, a capability that is difficult to achieve with single-modal sensors. Naturally, this performance enhancement comes at the expense of simplicity. Each sensing modality possesses unique analyte detection capabilities; through synergistic effects, they enhance signal response, broaden the detectable analyte range, and elevate overall analytical performance. A low limit of detection reflects analytical sensitivity under optimized conditions and does not necessarily indicate better clinical diagnostic performance. When evaluating clinical samples, parameters such as sample number and type, reproducibility, recovery, comparison with reference methods, clinical sensitivity and specificity, and independent validation are at least equally important. Therefore, dual-modal and multi-modal sensors should be assessed not solely by their LOD, but also by their performance in real clinical matrices. The main advantage of dual-modal and multi-modal sensing is its potential to improve analytical reliability and resistance to interference through signal cross-validation (Table 1 and Table 2). The data reflect analytical performance only and cannot be directly used to compare clinical diagnostic value.

Compared with single-modal sensing, dual-modal or multi-modal sensing offers advantages such as improved sensitivity, enhanced reliability, better anti-interference capability, and expanded analytical dimensions. For example, a smartphone-integrated fluorescence/absorbance dual-modal platform achieved self-calibration through dual-signal cross-validation, with RSD values of 5.6% and 0.8% across multiple electrodes, showing clear improvement in reliability [168]. A dual-CRISPR/Cas12a-based tri-modal biosensor effectively distinguished single-base mismatched miRNA through three independent signal channels, representing a substantial improvement in selectivity over single-modal methods [166].

While single-modal sensing platforms are irreplaceable for cost-effective, simple-operation testing, multi-modal sensors, which integrate information fusion and complementary technical merits, possess remarkable capacity for complex sample analysis and ultrasensitive detection. In practical applications, the choice must be rationally made based on target complexity, budgetary constraints, and performance requirements, or by exploring simplified multi-modal designs to balance performance and cost. The construction of multi-modal systems typically involves complex nanomaterial synthesis and interface modification.

As sensing modes evolve from single-modal to dual-modal and then to multi-modal, the overall sensing capability and analytical reliability tend to improve, although detection efficiency and method reproducibility may gradually decline. Single-modal sensors are simple and low-cost. Dual-modal sensors strike a good balance between sensing capability and analytical reliability, and show relatively high potential for clinical translation. Multi-modal sensors offer the highest analytical reliability, but their construction often involves complex material synthesis and sensing-interface modification, which can compromise detection efficiency and reproducibility. Furthermore, due to the integration of diverse optical and electrical components, achieving high device integration and portability presents formidable engineering challenges, which remain a primary focus for researchers in the field. When testing clinical samples, factors such as sample number and type, method reproducibility, and detection time need to be comprehensively considered. Compared with single-modal and multi-modal sensing, dual-modal sensing currently provides strong overall sensing capability, good analytical reliability, and shorter detection times, giving it relatively high potential for clinical translation.

## 7. Towards Clinical Translation

### 7.1. Current Technical Bottlenecks

Although sensing technologies have delivered outstanding performance and great promise at the laboratory scale, their translation into clinical practice is impeded by a series of critical hurdles. First, complex biological matrices contain abundant interfering components that demand labor-intensive pretreatment steps before measurement, an issue that severely restricts the implementation of point-of-care testing. For this reason, fully integrated sample-in-result-out analytical systems have become an indispensable developmental target, regardless of the superior sensitivity achieved by state-of-the-art sensors. Second, although point-of-care testing systems show promising potential for tumor biomarker detection, some of them still require relatively complex material synthesis steps (e.g., solvothermal synthesis of MOFs, multi-step modification of nanoparticles), specialized instruments (e.g., spectrometers, electrochemical workstations), or complicated preparation procedures, restricting their practical deployment in genuine POCT scenarios. For instance, while SERS-based sensing platforms offer extremely high sensitivity, the batch-to-batch reproducibility of SERS substrates remains a key bottleneck for their clinical translation.

Furthermore, single-modal, dual-modal and multi-modal sensing architectures all suffer from distinct engineering and application limitations: poor anti-interference performance of single-modal sensors, difficult assembly of dual-modal integrated devices, excessive manufacturing costs and inferior interfacial stability for multi-modal platforms. Signal crosstalk constitutes another prominent technical obstacle to multi-modal sensing. Signal crosstalk refers to unintended interference from the excitation or output signal of one sensing modality with the detection process of another. Its sources can be broadly grouped into material, interfacial, physical, and signal-processing aspects. For instance, excitation illumination adopted in optical characterization can perturb the background current of electrochemical electrodes. In turn, local microenvironment variations induced by electrochemical redox reactions are capable of quenching optical response signals. Such reciprocal interference imposes strict design specifications to achieve orthogonal, crosstalk-free acquisition of multiplexed sensing outputs. To tackle this issue, researchers have explored several signal-isolation strategies. One study, for instance, coupled a precisely controlled non-Faradaic potential window with time-resolved acquisition, enabling synchronous but independent readout of electrochemical and optical signals without perturbing the spectral background [169]. Gao et al. [170] built a cross-modal logic-gate sensing platform using a proximity ligation strategy, assigning independent output channels to different targets to help prevent signal crosstalk and mutual interference. Another group developed a ratiometric PEC-EC aptasensor that translates one recognition event into two opposite signal outputs; the ratio between them offers built-in self-calibration that can help reduce environmental interference [171]. In addition, most tumor-associated biomarkers exhibit insufficient disease specificity, as their aberrant expression occurs in a wide spectrum of malignancies as well as benign pathological lesions. This inherently raises the probability of misdiagnosis when diagnosis is based solely on one or a limited panel of biomarkers. Collectively, all the above drawbacks constitute the core last-mile barriers that hold back the clinical transformation and large-scale practical deployment of high-performance biosensing platforms.

### 7.2. Frontier Directions

To meet the demands for on-site diagnostics, sensors are evolving toward miniaturization and integration, relying on the optimization of microelectronics and circuit design alongside collaborative innovations across multiple technologies. Portable biosensors enable rapid, precise, and decentralized detection of cancer biomarkers, facilitating disease management from the source. These systems typically utilize colorimetric or thermal signal outputs, readable via smartphones or handheld instruments, offering user-friendly operation and cost-effectiveness. Such advancements significantly reduce patient visits and waiting times, particularly in resource-limited settings, thereby enhancing the accessibility of high-quality diagnostic services. Future biosensors will transcend their role as mere signal collectors to become comprehensive data processing terminals. By integrating AI-driven machine learning algorithms, high-dimensional datasets acquired from multi-modal sensors can be systematically analyzed to identify latent feature patterns and subtle diagnostic signatures that are indiscernible via manual observation, thereby facilitating early disease diagnosis and longitudinal monitoring of disease progression. Furthermore, integrating patient-specific data can assist in formulating personalized therapeutic regimens and benefit broader populations through remote surveillance. Machine learning models in AI-enhanced multimodal sensing frequently grapple with high-dimensional, noisy data burdened by batch effects. Principal component analysis (PCA) and partial least squares discriminant analysis (PLS-DA) remain standard tools for deconvolving overlapping SERS spectra or multichannel electrochemical currents into interpretable latent variables, facilitating effective dimensionality reduction and discrimination in sensor array classification. In one representative study, Song et al. [172] combined an oxygen-deficient MoO_3-X_ SERS substrate with a PCA-LDA model to achieve three-class classification of hepatoma cells, esophageal cancer cells, and leukocytes. This approach yielded an accuracy of 92.22%, with 90% accuracy for esophageal cancer subtyping and an AUC of 0.97. For non-invasive cancer screening, Feng et al. [173] applied PLS-DA to the SERS spectra of urinary modified nucleosides, obtaining diagnostic sensitivities of 95.2%, 90.9%, and 98.1% for nasopharyngeal cancer, esophageal cancer, and healthy controls, respectively. Beyond classification tasks, artificial neural networks have also been utilized to optimize biosensor fabrication parameters. Imani et al. [174] applied an ANN-GA framework to capture how fabrication parameters and output current are nonlinearly related in plasmonic miR-155 detection, achieving an R^2^ of 0.9845 and effectively minimizing the need for iterative experimentation. Despite these advances, practical implementation still faces notable hurdles, including limited training datasets, insufficient model interpretability, and a lack of independent clinical validation. Addressing these gaps through standardized datasets and external validation will be crucial for enhancing clinical credibility.

## 8. Conclusions

The precise detection of tumor biomarkers is the cornerstone for reducing cancer mortality and achieving early diagnosis and intervention. Biosensing represents a vibrant, interdisciplinary frontier. With advancements in biotechnology and nanomanufacturing, the profound integration of nanomaterials and sensing methodologies will continue to drive breakthroughs in detection sensitivity and multifunctional integration. This review has systematically delineated the evolutionary trajectory of biosensing technology from conventional single-modal signal readouts to sophisticated multi-modal synergistic analysis. While single-modal sensors have played a significant role in fundamental screening, they remain limited in handling complex biological matrices and ultra-trace targets. In contrast, multi-modal biosensors, by integrating the high sensitivity of electrochemistry with the superior specificity of optical modalities, can provide conditional signal cross-validation and self-calibration when the signal-generation processes are sufficiently independent, thereby catalyzing a paradigmatic shift in the biosensing landscape.

After decades of rigorous research, the development of integrated sensing platforms for the rapid and accurate detection of cancer biomarkers has garnered global attention and is poised to become a promising diagnostic strategy in the future of precision medicine. Looking ahead, the effective fusion of multi-modal sensors with AI-driven data analytics is expected to further extend the boundaries of biomarker detection in precision diagnostics and real-time monitoring. However, the construction of such multimodal platforms often involves complex material synthesis and sensing interface modification, which can reduce detection efficiency and reproducibility. At the same time, the practical translation of multi-modal sensing is constrained by multiple challenges, including cumbersome component integration, complicated signal data analysis, limited device portability, the absence of unified fabrication and testing protocols, high costs caused by sophisticated nanomaterials and precision instruments, unresolved technical bottlenecks for scale-up manufacturing, stringent regulatory requirements for in vitro diagnostic devices, and incomplete validation based on independent clinical cohorts. The next generation of biosensors will evolve toward higher levels of integration, intelligence, and precision. This will require not only the resolution of engineering hurdles such as signal crosstalk and manufacturing consistency but also the cross-disciplinary synergy of chemistry, materials science, medicine, analytical science, and computer science. It is anticipated that high-performance multi-modal sensing platforms will ultimately transcend laboratory constraints and serve as robust tools for early cancer screening and personalized tumor clinical management.

## Figures and Tables

**Table 1:** Representative performance of Single-modal biosensors for tumor biomarker detection.

Method	Analyte	Sensing Material	Matrix	Linear Range	LOD	Construction Time	Selectivity	REF
EC	CEA	AuNPs/PCL-b-PHEAA	serum	100 fg/mL–200 ng/mL	60 fg/mL	9.5 h	Vs. CYFRA21-1, HER2, cTnI	[59]
	CEA CA72-4	AuNPs-TB-GO/AuNPs- FMC-WS2	serum	0.01–120 ng/mL 0.05–35 U/mL	0.003 ng/mL 0.016 U/mL	overnight + 2 h	Vs. BSA, CA211, ABCB1, CA19-9	[62]
CL	MUC1	AuNSs-PLA-PEG-Apt	serum	500–20,000 cells/well	450 cells/well	0.33 h	Vs. High specificity for Muc1-positive cells	[70]
	CA125 CEA CA19.9 CA15-3	bi-functionalized AuNPs	serum	10.0–200.0 U/mL 10.0–150.0 U/mL 10.0–250.0 U/mL 20.0–200.0 U/mL	5.3 U/mL 4.6 U/mL 5.7 U/mL 14.3 U/mL	0.05 h	Vs. Hemoglobin, albumin, triglyceride	[71]
	BRCA1	CRISPR/Cas12a	serum	0.05–100 nM	30 pM	1 h	Vs. BRCA2, HPV16, HPV18, HBV	[73]
FL	CA125	Nano-GO	serum	0.1–10 U/mL	0.01 U/mL	0.67 h	Vs. High fluorescence intensity for target proteins	[86]
	HE4 CEA AFP			10–100 pg/mL	1 pg/mL			
	CEA AFP	NESA	serum	1–500 ng/mL 5–800 ng/mL	0.7 ng/mL 2 ng/mL	3.3 h	Vs. SCD146, lgG, BSA	[87]
SERS	TNF-α	Au@4-MBN@Ag	serum	10^−12^–10^−5^ mg/mL	4.37 × 10^−15^ mg/mL	2 h	Vs. BSA, Glu, lgG, IL-6, PCT	[97]
	CEA	AgPb	serum	1 pg/mL–1000 ng/mL	0.41 pg/mL	2 h	Vs. AFP, PSA, Lys, GLT, HFABP, CRP, HAS, GLB, ALB	[99]
PEC	CYFRA21-1	Bi_2_S_3_/Bi_2_O_2_(OH)_2_	serum	50 fg/mL–100 ng/mL	17 fg/mL	5 h	Vs. PSA, TPS, AFP, CEA	[117]
	CEA	BVO/BNFO	serum	1 pg/mL–0.1 mg/mL	1.91 pg/mL	13.5 h	Vs. PSA, CA125, AFP, CA19-9	[118]

**Table 2:** Performance comparison of representative dual-modal and multi-modal sensing platforms.

Mode	Method	Analyte	Sensing Material	Matrix	Linear Range	LOD	Construction Time	Selectivity	REF
Dual-mode	EC-CL	PD-L1	Ag@MNPs	serum	10^3^–10^9^ particles/mL (EC) 10^6^–10^9^ particles/mL (CL)	37.2 particles/mL (EC) 612.6 particles/mL (CL)	5 h	Vs. HIS, Glu, IgG, ERBB2	[124]
		MCF-7	MoS_2_-Au@Pt nanozyme	serum	10–10^9^ particles/mL (EC) 10^4^–10^9^ particles/mL (CL)	9.3 particles/mL (EC) 4200 particles/mL (CL)	Overnight+1.58 h	Vs. MCF-7, HepG2, HeLa, Hs578Bs	[125]
		microRNA-21	GDY-Gr	serum	0.1–10,000 fM (EC) 0.1 pM–1 nM (CL)	0.333 fM (EC) 32 fM (CL)	Overnight+2 h	Vs. NC, smRNA, tmRNA, miRNA-141/199a/155	[127]
	EC-FL	HER2	ZIF-67@Sb	serum	0–10 pg/mL	3.4 fg/mL (EC) 6.9 fg/mL (FL)	0.5 h	Vs. HER2, ER	[131]
		ER			0–10 pg/mL				
		CEA	QDLISA/Cd QDs	serum	0–320 ng/mL (FL) 0.01–20 ng/mL (EC)	0.18 pg/mL (FL) 0.003 pg/mL (EC)	2 h	Vs. CA125, CA19-9	[132]
		GP73	MoS_2_-Fc-PdNPs/N-GQDs	serum	1.00–10.0 ng/mL	0.0425 ng/mL (EC) 0.812 ng/mL (FL)	1.67 h	Vs. AFP, IgG, GPC3, HSA	[133]
	EC-SERS	PSA	Au@MoS_2_/SPE, TMB/Ab2/AuNPs	serum	0.005 ng/mL–50 ng/mL	3.58 pg/mL (EC) 4.83 pg/mL (SERS)	1.67 h	Vs. BSA, CEA, AF	[144]
		miR-106a	MoS_2_/AgNRs	serum	100 fM–100 nM	248.01 fM (EC) 67.44 fM (SERS)	Not explicitly stated	Vs. SM, miR-155, miR-21	[22]
		microRNA-21	Au NPs/FTO	serum	5 fM–100 pM (EC) 0.5 fM–0.5 pM (SERS)	2.2 fM (EC) 0.12 fM (SERS)	1.33 h	Vs. SM, miR-210/155/126	[141]
	EC-PEC	HER2	BiOBr_0_._8_I_0_._2_/CoSx	serum	0.005–15 ng/mL (EC) 0.001–10 ng/mL (PEC)	1.06 pg/mL (EC) 0.31 pg/mL (PEC)	Overnight+3 h	Vs. CA125, HE4, CEA, Sarcosine	[152]
		HE4	Au NPs/CdS NS	serum	0.01–200 ng/mL (EC) 0.01–100ng/mL (PEC)	1.188 pg/mL (EC) 1.084 pg/mL (PEC)	0.23 h	Vs. MUC1, CEA, CYFRA21-1, PCT	[153]
		miRNA-133a	Cu-TCPP/BiVO_4_	serum	0.1 fM–1 pM	0.02 fM (EC) 0.003 fM (PEC)	6.5 h	Vs. miRNA-499, Lactic acid, Ascorbic acid, Uric acid, Glucose, cTnI, miRNA-133a	[154]
		CYFRA21-1	PTCDA@C_60_/AuNPs	serum	0.001–40 ng/mL (EC) 0.0001–4 ng/mL (PEC)	0.3 pg/mL (EC) 0.03 pg/mL (PEC)	11.5 h	Vs. VEGF165, CEA, CA125, NSE	[20]
Multi-mode	PEC-CL-photothermal	PSA	TiO_2_/ZIF-8/Cu(II)	serum	10^−7^–10^−12^ g/mL	0.16 pg/mL	6.42 h	Vs. BSA, CEA, AFP	[156]
		CEA	3D hollow HCdS@Au	serum	0.015–2.4 ng/mL	3.5 pg/mL	1.58 h	Vs. BSA, Gox, EXO	[157]
	EC-CL-FL	HER2	Cu-MOF/aptamer	serum	4.5 fg/mL–20 ng/mL	1.51 fg/mL 9.19 fg/mL	2.67 h	Vs. PR, Ki67, CEA, PSA	[158]
		ER			10 fg/mL–20 ng/mL				
	CL-FL- SERS	microRNA-21	UCNPs/Au-DTNB@A	Saliva, serum	1 fM–2 nM	1 pM	0.33 h	Vs. miR-141/155, NS	[159]

## Data Availability

Available on request from the corresponding authors of the data presented in this review.
